# Postprandial Responses to Animal Products with Distinct Fatty Acid and Amino Acid Composition Are Diet-Dependent

**DOI:** 10.3390/nu17091581

**Published:** 2025-05-04

**Authors:** Bjørg Egelandsdal, Anna Haug, Jens F. Rehfeld, Sílvia Coutinho, Lledó Roglà Ricart, Harald Martens, Milena Monfort-Pires, Catia Martins

**Affiliations:** 1 Faculty of Chemistry, Biotechnology and Food Science, Norwegian University of Life Sciences, 1433 Ås, Norway; 2 Faculty of Biosciences, Norwegian University of Life Sciences, 1433 Ås, Norway; anna.haug@nmbu.no; 3 Department of Clinical Biochemistry, Rigshospitalet, University of Copenhagen, 1172 Copenhagen, Denmark; jens.frederik.rehfeld@regionh.dk; 4 Department of Nutrition, University of Oslo, 0316 Oslo, Norway; 5 Department of Nutrition, Food Sciences and Gastronomy, Torribera Food Campus, University of Barcelona, 08921 Barcelona, Spain; lledoroglaricart@gmail.com; 6 Department of Engineering Cybernetics, NTNU-Norwegian University of Science and Gløshaugen, O. S. Bragstad Plass 2/Idletechs AS, 7034 Trondheim, Norway; harald.martens@ntnu.no; 7 Turku PET Centre, University of Turku, 20520 Turku, Finland; mmopir@utu.fi; 8 Department of Nutrition Sciences, University of Alabama at Birmingham (UAB), Birmingham, AL 35233, USA; catia197@uab.edu

**Keywords:** postprandial study, appetite hormones, lipid quality, animal food

## Abstract

Background: Though evidence is limited, animal products like pork sausages and cheese may affect satiety differently due to their distinct protein, fat, and calcium content. This study therefore compared their acute effects on breakfast using appetite-related markers. Methods: A total of 11 women and 13 men, with a mean age of 23.0 ± 2.6 years and mean BMI of 24.5 ± 2.6 kg/m^2^, participated in this crossover design study. Concentrations of active ghrelin, glucagon-like peptide 1 (GLP-1), glucose-dependent insulinotropic polypeptide (GIP), cholecystokinin (CCK), insulin, glucose, leptin, and blood lipids were measured. Subjective feelings of appetite using visual analogue scales were analyzed (0–4 h) as a response to two test breakfasts meals with a similar energy and macronutrient content. Appetite feelings and energy intake from an ad libitum buffet lunch were subsequently measured. Data were analyzed using two different ANOVA methods. Results: The pork sausage breakfast was characterized by an earlier triglyceride (TG) peak than the cheese. A slower TG clearance was seen with the cheese breakfast. Ghrelin suppression was longer in the pork sausage breakfast. Active GLP-1 concentration was higher following the cheese breakfast and active GIP declined slower. The two ANOVA methods disagreed regarding the insulin effect. Subjective feelings of hunger before buffet and ad libitum energy intake were higher in males (791 ± 64 kcal) compared with females (344 ± 32 kcal), but did not differ between breakfast types. Conclusions: Acute consumption of pork and cheese of the same energy, fat, and protein content provided detectable differences in appetite-related hormones and lipid responses. Appetite and lipid metabolism were affected by the major differentiators of the test meals, namely calcium, fatty acids and amino acids compositions.

## 1. Introduction

Understanding the interplay between food and appetite regulation is useful for managing obesity and preventing associated metabolic disorders, such as type 2 diabetes, some types of cancer, and cardiovascular diseases [1,2].

Postprandial studies are considered valuable tools for examining the transient effects of food products on circulating cardiovascular disease risk markers, such as glucose and triglyceride concentrations [3]. Such studies are important, not only in the context of metabolic disease prevention, but also for food product development [4,5]. Postprandial appetite-related hormones’ analyses are also pivotal to understanding the effects of different food products on the short-term control of appetite [6].

When analyzing the effects of macronutrients on satiety, the consensus is that lipids are less satiating than proteins and carbohydrates [7,8]. The effects of carbohydrates on satiety depend on their structure and content (i.e., fibre, solid carbohydrates, etc. [9]; however, the latter becomes irrelevant when animal products are studied. On the other hand, the postprandial effects of different fatty acids—such as saturated fatty acids (SFA), monounsaturated fatty acids (MUFA), and polyunsaturated fatty acids (PUFA)— are less clear, especially regarding their effects on hunger suppression, even though most studies indicate that PUFAs, followed by MUFAs, result in greater hunger suppression (indicated by lower active ghrelin plasma concentrations) than SFAs [10]. Strik et al. [11] found no significant differences in subjective feelings of appetite or in plasma concentrations of appetite-related hormones after the consumption of three high-fat meals with different fatty acid compositions (SFA, MUFA, and PUFA). Similarly, Rust et al. [12] did not observe differences in ghrelin secretion after meals with varying fatty acid compositions. The postprandial appetite effects of different protein sources have also been investigated. The proposed underlying mechanisms for elevated satiety after protein intake include, among other factors, a differential increase in the secretion of satiety hormones such as active glucagon-like peptide-1 (GLP-1), cholecystokinin (CCK), and peptide YY (PYY) (Reviewed by Moon J and Koh G [13]), and their potential to influence gastric emptying and gastrointestinal motility [14]. The satiety response regulating factors of protein related to amino acid compositions are less clear.

Furthermore, nutrients like calcium have also been reported to reduce subjective appetite feelings [15]. Even though the evidence regarding the impact of calcium on the release of appetite-related hormones remains inconclusive [15,16,17], a recent study showed that intraduodenal infusion of 1000 mg of calcium in young males with normal body weight increased plasma concentrations of CCK, GLP-1, and PYY [18]. 

Animal products like pork and cheese recently demonstrated metabolic benefits when part of a healthy dietary pattern [19]. Pork and dairy products differ in their amino acid profiles, fatty acid compositions, and calcium content. Many animal food proteins are complete or nearly complete in terms of amino acid composition, which may limit variation in satiety and satiation, as well as in feelings of hunger and fullness. However, it may be hypothesized that differences in fatty acids, particularly when the protein-to-fat ratio is low, may have a greater impact on satiation than small differences in protein composition. 

Our understanding of the satiety and satiation mechanisms associated with different animal products remains limited, especially when comparing food items with similar macronutrient distribution [20]. Furthermore, few studies have examined how animal products with distinct amino acid and fatty acid compositions affect appetite and postprandial metabolism [21,22].

This study aimed to compare the acute effects of consuming two breakfast meals with the same energy and macronutrient distribution, one with Gouda-type cheese and the other with pork breakfast sausages, on the plasma concentration of appetite-related hormones, subjective feelings of appetite, and lipid/glucose profiles. The primary research hypothesis was that there would be differences in appetite makers following the ingestion of cheese versus pork sausage induced by fatty acid.

## 2. Materials and Methods

### 2.1. Study Design

**Figure 1:**
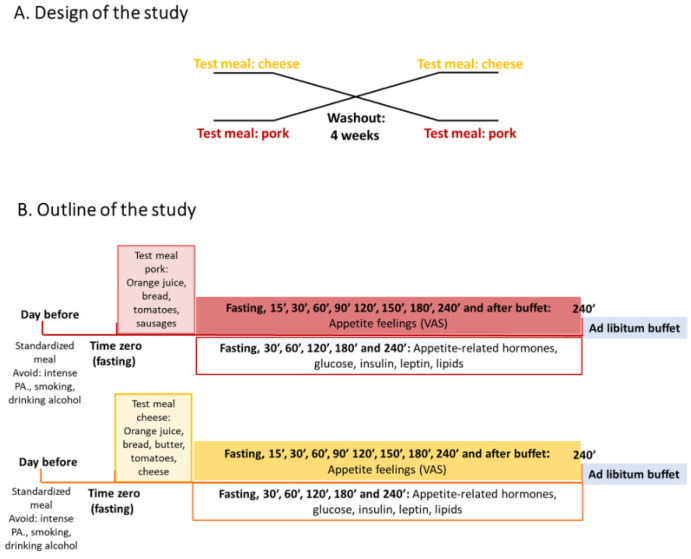
(**A**) Study design: a cross-over postprandial study examining two test products (cheese and sausage). (**B**) Outline of the study: The test products were provided in a breakfast meal in random order. Blood sampling was performed from fasting up to 240 min and, afterwards, an ad libitum buffet lunch was provided, where both appetite feelings and energy intake were measured. Visual analogue scales (VAS) were used to assess subjective ratings of appetite throughout the study period. Energy intake was measured using the Norwegian Directorate of Health and Norwegian Food Safety Authority online dietary tool (*Kostholdsplanleggeren, in Norwegian* [23]).

This study used a randomized crossover design, with a 4-week washout period, as outlined in Figure 1. Gouda-type cheese- and pork sausage-containing breakfasts were consumed. Blood was drawn in the fasting state and postprandially for 240 min to assess plasma concentrations of appetite-related hormones (described below), leptin, insulin/glucose, and lipids. In parallel, visual analogue scales (VAS) were used to assess subjective feelings of appetite. Four hours after breakfast, participants were offered an ad libitum buffet lunch, and energy intake and appetite feelings were subsequently measured.

### 2.2. Participants

Participants were recruited at the Norwegian University of Life Sciences (NMBU) in Ås, Norway. The recruitment was carried out with the help of internet advertising and posters distributed at the university campus. The inclusion and exclusion criteria are the same as previously described [19]. In summary, those who did not consume meat and/or dairy products, were trying to lose weight, or had food allergies, were excluded. 

### 2.3. Breakfast Test Meals

Composition: Table 1 and Table 2 show the content and macronutrient distribution of the two breakfast meals, respectively. The breakfast meals provided 28% of the total daily energy requirements, calculated using the Norwegian Government online tool Kostholdsplanleggeren to the nearest 100 kcal [23]. The aim was to provide participants with a meal close to a standard breakfast in Norway. Most Norwegians (81%) [24] consume bread for breakfast, and about half of them choose cheese as a topping [25]. Pork meat at breakfast is traditionally eaten as bacon, cooked ham or sausages.

The participants were given more sausages (152 g) than cheese (104 g) for their breakfasts (Table 1). This compensated for the fact that macronutrient differences in the test products: protein, total fat, and energy content per 100 g were significantly different (*p* < 0.05) for the two test products (Table 2). The test samples (cheese and sausage) were the main sources of fat and protein in the breakfast test meals. The breakfast was a reduced carbohydrate meal, as the carbohydrate content was below the daily recommended level [26]. The breakfast meal contained 540 kcal, with 47% of energy provided from fat, 32% from carbohydrates and 21% from protein (Table 1). The cheese was a commercial Gouda-style cheese produced by TINE. The pork breakfast sausage was produced “in-house” and production details are provided in Supplementary Materials. To obtain the same amount of fat and protein from each test diet, butter was used for balancing fat. A 2.1% reduction in total PUFA was calculated when butter was used to obtain the same amount of total fat from dairy and pork.

The cheese did not contain carbohydrates or sugars and the pork meat and fat contained 0.1 g carbohydrates/100 g [27]. The calcium content in the test products was 0.82 g/100 g for cheese and 0.004 g/100 g for sausage, respectively [27,28].

### 2.4. Fatty Acids and Amino Acids Analysis of the Test Products

Fatty acid analysis: Samples were analyzed by gas chromatography as previously described [29]. In brief, methylated fatty acids were subjected to an 8890 GC system with a flame ionization detector (Agilent Technologies, Santa Clara, CA, USA). Separations were performed on a Varian CP7421 (200 m × 0.25 mm i.d.) column from Varian Inc. (Palo Alto, CA, USA). Identification was made by a fatty acid standard mixture (37 FAME-mix, Supelco, and 1269119 USP FAME standard mixture, Sigma-Aldrich, Oslo, Norway).

Table 3 shows the fatty acid composition of the test products. The sausage exhibited a higher proportion of MUFAs and PUFAs than the cheese, which contained more medium- and long-chain SFAs.

Amino acid analysis: Hydrolyzed samples were analyzed by ion exchange chromatography, as previously described [30]. The Biochrom 30+ Amino Acid Analyzer system (Biochrom Ltd., Cambridge, UK) was used. Amino acids were determined by a reaction with ninhydrin and photometric detection at 570 nm (440 nm for proline). A high-pressure column packed with Ultropac 8 cation exchange resin (Biochrom Ltd., Cambridge, UK) was used. The Biochrom 30+ data handling software (Chromeleon^TM^ 7.3 CDS) determined the individual amino acid. Some amino acids measured in the three major protein sources used in the breakfast meal are shown in Table 4.

### 2.5. Anthropometric Measurements

Anthropometric measurements were taken in the fasting state using standardized procedures. Height was measured in cm with one decimal place using a portable stadiometer (Charder HM200P Portstad, Taiwan). Weight was measured in kg with one decimal place using a Tanita TBF-300A scale (Sindelfingen, Germany). 

### 2.6. Outcome Variables

Appetite-related hormones: Blood samples were collected in 4 mL EDTA-coated tubes and drawn at fasting, and every 30 min up to 3 h; the last measurement at 4 h. For ghrelin, GLP-1, GIP, and leptin, 1 mL of whole blood was transferred into a microtube and a 20-μL mixture of inhibitor (10 μL of Pefabloc [Roche Diagnostic] + 10 μL of dipeptidyl peptidase IV inhibitor [Merck Millipore], Burlington, MA, USA) was added. For CCK, 500 KIU of aprotinin (DSM, Coatech AB, Engelviken, Sweden) per mL of whole blood was added to the EDTA-coated tubes. Samples were then centrifuged at 2106 relative centrifugal force (RCF) for 10 min at 18 °C and the plasma frozen at −80 °C until further analysis. Ghrelin, GLP-1, and GIP were all determined in their active states, meaning after a specific post-translational change in the molecules.

Plasma samples were analyzed for ghrelin, GLP-1, GIP, and leptin using a Human Metabolic Hormone Magnetic Bead Panel (HMHEMAG-34 K, Merck KGaA, Darmstadt, Germany). Cross-reactivity between antibodies and any of the other analytes in this panel is undetectable or negligible. CCK was analyzed using an “in-house” radioimmunoassay [33]. Intra- and inter-assay coefficients of variation were <10% and <15% for ghrelin, GLP-1, GIP, and leptin; and <5% and <10% for CCK, respectively. All the samples from the same participant were analyzed in the same plate. The analyses of ghrelin, GLP-1, GIP, and leptin were performed by the same technician at the Norwegian University of Science and Technology (NTNU). CCK was analyzed at the University of Copenhagen, Denmark. 

Markers of lipid and glucose metabolism: All serum samples were sent to Fûrst Medical Laboratory (https://www.furst.no/, accessed on 6 January 2022) for the measurements of TG, total, LDL, and HDL cholesterol, insulin, and glucose using accredited methods.

Subjective appetite feelings: Subjective feelings of hunger, fullness, desire to eat, and prospective food consumption were assessed using a 10 cm VAS in the fasting state, immediately after the consumption of the breakfast meals, at 15’, 30’, 60’, 90’, 120’, 180’, 240’, and after the ad libitum buffet lunch, as previously described [34]. A composite average appetite score was calculated as follows: Appetite score (mm scale) = [desire to eat + hunger + (100 − fullness) + prospective consumption]/4 [35].

Ad libitum buffet lunch: Four hours after breakfast consumption and at the end of the blood sampling period, participants were offered an ad libitum sandwich-buffet lunch and asked to eat as much or as little as they wanted until comfortably full. The toppings were selected based on the participants’ preferences. During screening, participants were asked to rank four sandwich toppings: cheese, ham, liver pate, and mackerel in tomato sauce, in order of preference and were then given the second or third choice during both test days. Participants were given water to drink (ad libitum) and had to eat alone in a separate room. Each sandwich had the same energy content, as well as fat and protein content. The sandwiches were weighed before and after the participant finished eating. The participants were given sandwiches cut into quarters (totalling 6 slices for females and 10 slices for males).

### 2.7. Power of Study

The sample size required to find differences in the plasma concentration of appetite-related hormones and subjective appetite feelings between the two breakfast meals was regarded as adequate based on previous literature [6,36,37]. The study was underpowered to look at differences in an ad libitum buffet energy intake between the two test-meals [38], but the ad libitum experiment was nevertheless performed.

### 2.8. Statistical Analysis

General Linear Models using basal (time zero) measurements with subjects added [Repeated measure (RM)-1 factor ANOVA]; *t*-tests and Z-tests were calculated in MINITAB 18 (Minitab, LLC, 2021, Minitab, Lock Haven, PA, USA). Measured circulating concentrations of biomarkers were subjected to a repeated measure RM-2- factor ANOVA, with time points and meal type as independent variables and the various blood markers as dependent variables. Sex was added as a between-subject factor [39]. IBM SPSS Statistics for Windows, Version 27.0. (IBM Corp., Armonk, NY, USA) was used.

Direct comparisons of time series were made with or without transformation to a common scale. The following normalization procedures were tested: area, mean, range, max, peak, and unit vector normalization (Unscrambler^®^ X 10.4 &11, Aspen Unscrambler, Bedford, Massachusetts, USA). Max normalization (MN) was thereafter chosen since individual scale variation (due to sex) in blood markers seemed most optimally removed. The method of 50-50 MANOVA [40] was used [41] with the individual time series modelled with sex, diet, and their interaction effect diet * sex (D*S). Sex and subjects were defined as covariates. MN is sensitive to outliers [42]. Since the ghrelin analysis showed one clear outlier (from Grubbs test, MINITAB 18), this measurement was defined as missing.

Principal component analysis (PCA) and partial least squares regression (PLSR) analysis were performed in Unscrambler X and MATLAB (MATLAB, 2010, version 7.10.0 (R2010a), The MathWorks Inc., Natick, MA, USA). PCA was used to focus on the differences between the two breakfast meals (pork sausage—cheese). The scale adjustment method for PCA was performed by dividing each variable by its own standard deviation. This ensures that all variables have the same chance of contributing to co-variation patterns. However, to include the full effect difference between the two meals, mean cente ring of the normalized variables was not performed. Internal consistency in the data was used as tentative criteria for relationships on this small data set of 24 subjects instead of strict statistical distribution tests.

VAS data from 15 min to 240 min were also analyzed using 50-50 MANOVA. The method in reference [35] was used to merge all VAS variables before the statistical analysis. GraphPad Prism version 9.0 for Windows (GraphPad Software, San Diego, CA, USA) and Excel 365 (Microsoft Corporation, 2018, Redmond, WA, USA) were used for graphs. 

Data are presented as mean ± s.e.m or s.d.

## 3. Results

**Figure 2:**
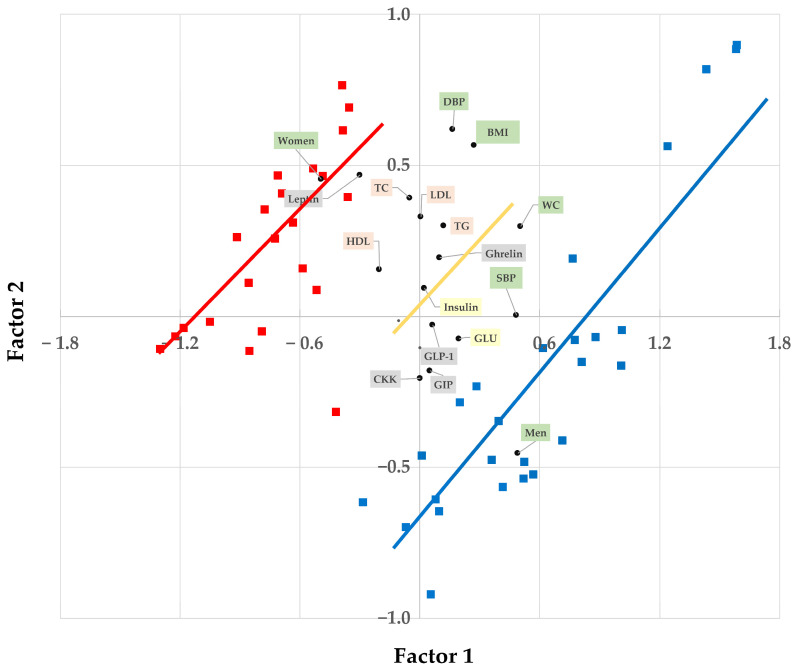
Schematic drawing of the individual variation using data from Table 5. Blue line and squares = men; red line and squares = women. The yellow line indicates the direction for basal variables with limited sex influence. Data (scores and loadings) were obtained from a partial least squares regression.

Twenty-five participants were recruited. Table 5 and Figure 2 provide the baseline characteristics of the 24 (13 men and 11 women, mean age 23.0 ± 2.6 years, age range 20–29 years) participants who completed the study. Sex clearly was causal for variation for some basal values, but in the orthogonal direction to sex only waist circumference (WC) appeared influential (Figure 2). All participants were healthy, and the mean BMI ranged from 20.6 to 30.9 kg/m^2^.

The data acquired are presented below in the following order: (i) RM-ANOVA for the basal values; (ii) RM-ANOVA and 50-50 MANOVA analysis (MN is added if the data were normalized) of the time changes and their link to sex and diet; (iii) the VAS variables’ timescales linked to sex and diet using the same statistical methods; and finally (iv) the ad libitum buffet data.

### 3.1. Blood Markers

Some of the blood variables of the enrolled participants showed significant (*p* < 0.05) sex dependence at fasting. These variables (Figure 2) were leptin (explained variance (EV) 45%), HDL (EV 17%), and glucose (EV 12%). RM- 1 factor ANOVA was used. Total cholesterol (TC) tended towards significance (*p* = 0.088). Including further covariates (like BMI) in the ANOVA analysis for CCK at fasting gave a significant sex effect (*p* = 0.044). 

To examine the time effects of diets, the approach was to use 2-factor RM-ANOVA and 50-50 MANOVA. As absolute values for blood markers were not influenced by diet and some basal values could be extensively influenced by sex, max normalization (MN) was added to our analysis of variance. The 50-50 MANOVA method used complete time profiles as dependent variables. Comparable output variables between 50-50 MANOVA’s sex (S_M_) and diet (D_M_) would thus often be RM-ANOVA’s factors sex (S)*T, S, diet (D)*T, and D, respectively. Most time profiles are presented as figures in Supplementary Materials (Figure S1).

#### 3.1.1. Serum Triglycerides, Serum Cholesterols, and Plasma Leptin Hormone

**Figure 3:**
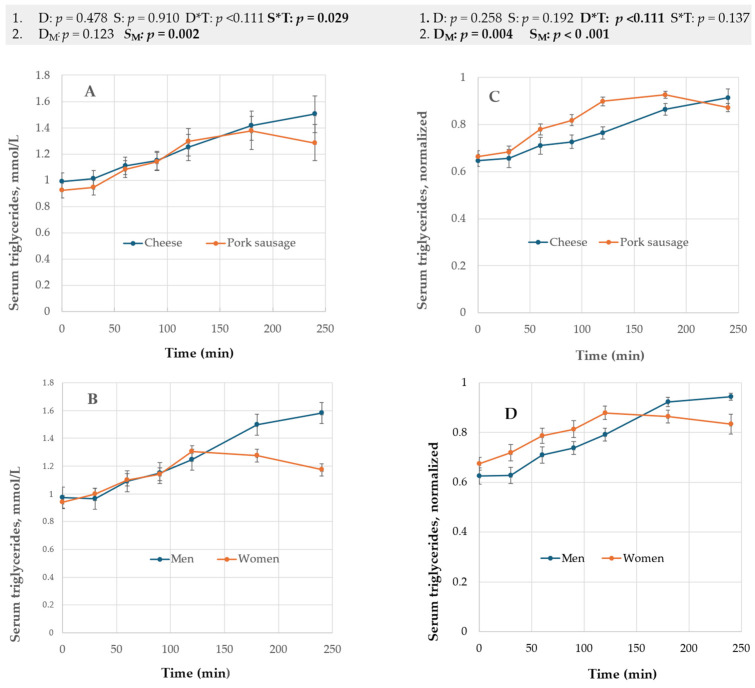
(**A**,**B**) show the average data for diet (panel A) and sex (panel B) used for RM-ANOVA (1); (**C**,**D**) are normalized input curves to MN RM-ANOVA and MN 50-50 MANOVA (2). The statistical outputs (*p*-values) are listed above the figures for models 1 and 2 for diet- (D; D_M_) and sex- (S, S_M_) related factors. T = time. Errors are ± s.e.m.

**Figure 4:**
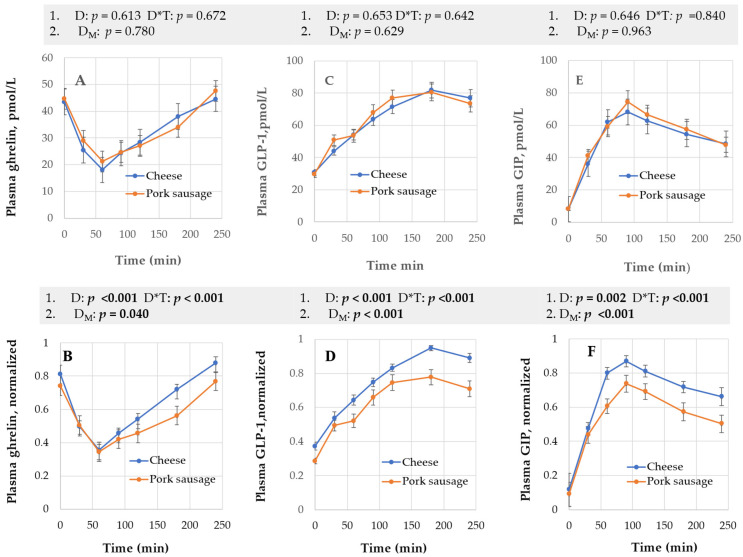
The panels (**A**–**F**) show the measured absolute time and their respective normalized curves for ghrelin, GLP-1, and GIP. *p*-values for selected factors, obtained using RM-ANOVA (model 1) and 50-50 MANOVA (model 2), are listed at the top of the figures. Errors are ± s.e.m.; D & D_M_ = diet; T = time.

TG: Serum TG declined numerically after 180 min for the pork sausage meal, while for the cheese meal, no serum TG maximum was observed within 4 h. Figure 3 and Figure 4 showed that selected markers (e.g., TG and GLP-1) may reach max at various times.

RM-ANOVA (raw data) identified S*T *(p* = 0.029) as significant (Figure 3A,B, Supplementary Materials and Table S2). MN RM-MANOVA supported a D*T effect (*p* < 0.001). MN 50-50 MANOVA supported a diet and sex effect (Figure 3C,D).

Total cholesterol (TC), LDL, and HDL: The time (T) changes for these variables were relatively small (Supplementary Materials and Figure S1, panels A–C). MN RM-ANOVA (normalized data used) suggested significant D*T factors for TC (*p* < 0.001) and LDL (*p* = 0.005) (Supplementary Materials, Table S2). In addition, TC diet was significant (*p* = 0.034, MN RM- ANOVA), presumably partly due to the reduction in S effects following normalization. MN 50-50 MANOVA regarded the D_M_ effects for all cholesterols as too small (EV < 2%) for significance (Supplementary Materials, Table S3). The 50-50 MANOVA method indicated that HDL was sex-dependent (*p* = 0.049), but no method using MN processed data supported this (Supplementary Materials, Tables S2 and S3). 

The cholesterol data, in particular the D-related outputs (D, D*T, D_M_) for TC and LDL depended on the statistical method used. This will be discussed below. 

Leptin: As expected from Figure 2, RM-ANOVA identified S (*p* < 0.01) and S*D (*p* = 0.031) as well as S*T (*p* = 0.039) as significant (Supplementary Materials, Table S2 and Figure S1, panel D). S*D and S were quite large factors as observed from EV. The 50-50 MANOVA method supported S_M_ as a large factor (*p* < 0.001) (Supplementary Materials, Table S3). Normalization removed sex as a significant factor and D*T became significant (*p* < 0.001) using MN RM-ANOVA; D_M_ was still identified as a too small factor for significance (Supplementary Materials, Table S3).

#### 3.1.2. Plasma Ghrelin, Active GLP-1, and GIP

The time changes for ghrelin, GLP-1, and GIP depending on diet are given in Figure 4. The comparable time profiles for sex are provided in Supplementary (Figure S1).

Ghrelin: RM-ANOVA suggested different responses between men and women (*p* < 0.001) (Supplementary Materials, Table S2). Ghrelin values at fasting were not significantly sex-dependent. Ghrelin plasma concentrations were numerically lower for pork from 2 to 3 h and the pork product somewhat delayed the increase in this hunger hormone (Figure 4A,B). MN RM-ANOVA provided significant D and D*T (both *p* < 0.001) and for MN 50-50 MANOVA, significant D_M_ (*p* = 0.040) and S_M_ (*p* = 0.034) (Supplementary Materials, Tables S2 and S3).

GLP-1: As expected, GLP-1 increased following meal intake RM-ANOVA (Figure 4C,D); max normalized (MN) raw data gave significant D, D*T, and D_M_ (Figure 4C,D and Supplementary Materials, Tables S2 and S3). Cheese then increased plasma GLP-1 more than sausages (Figure 4). Regarding sex, RM-ANOVA S*T (*p* = 0.041) and 50-50 MANOVA’s S_M_ (*p* = 0.051) indicated that males and females were different (Figure 4C). MN 50-50 MANOVA supported sex still being influential (S_M_, *p* = 0.022). Males had visually higher GLP-1 plasma concentration than females after 180 min (Figure 4D).

GIP: Absolute data gave no diet effect (Figure 4, panel E). MN RM-ANOVA identified D and D*T as significant (*p* = 0.002, *p* < 0.001, respectively). MN 50-50 MANOVA identified D_M_ as significant (*p* < 0.001) (Figure 4, panel F). GIP was more strongly expressed when cheese was the test product. Independent of the statistical method used, sex did not influence GIP’s time profile.

#### 3.1.3. Serum Insulin, Glucose, and Plasma CCK

**Figure 5:**
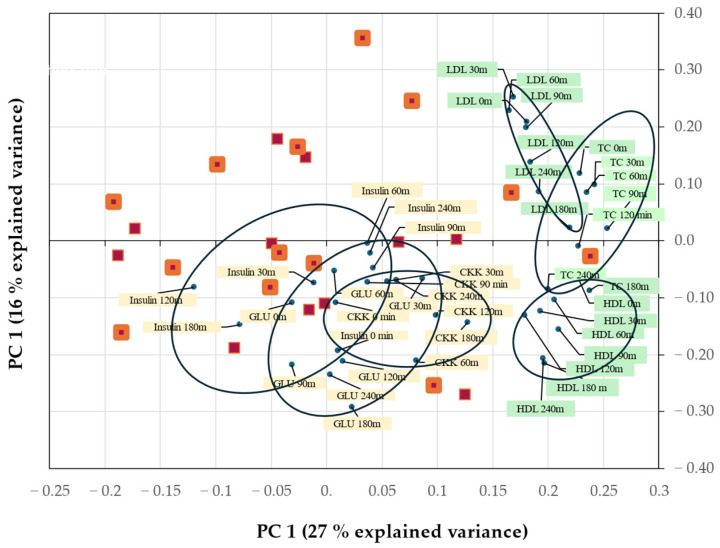
An exploratory PCA biplot showing how the main covariation patterns between the 24 subjects and meal differences (pork sausage–cheese) are related to six chosen variables, namely the three cholesterols (green) and three insulin/glucose/CCK variables (yellow) at their seven time points. As inputs, the absolute meal differences, with each of six variables × 7 time points normalized to a comparable scale, were used. Two PCs showed predictive validity in leave-one-person-out cross-validation. Squares are subjects; larger squares are men. Along each PC axis, zero means “no meal difference”, positive values along PC1 or PC2 suggest that pork sausage effects > cheese effects, and negative values means the opposite. Total explained variance is relative to data in Tables S2 and S3 (Supplementary).

RM-ANOVA suggested no significant diet factor (Supplementary Materials, Table S2). However, MN RM-ANOVA suggested that insulin clearly depended on diet for D and D*T (both *p* < 0.001, Supplementary Materials, Table S2). The 50-50 MANOVA (with or without MN) did not support this. Glucose, and to some extent CCK, gave similar results as insulin. As mentioned above, TC and LDL were variables where only MN RM-ANOVA gave statistically significant effects for D*T for LDL; TC also for D. Due to the disagreement, the above variables were examined further in a PCA biplot (Figure 5) using the difference in the variables between pork sausage and cheese (Ps-C) for each subject. The clear relation of TC to diet differences (PC1) agreed with MN RM-ANOVA that identified TC as a small explanatory factor for diet (D) (EV% = 3.8). For the smaller factor PC2, a systematic contrast between LDL on one hand and HDL, glucose, CCK, and to a limited extent TC, on the other hand, was observed. The 24 subjects were scattered. Insulin showed some co-variation with the two PCs, but it was small and less systematic, suggesting insulin as a small or unclear factor.

### 3.2. VAS Scale

**Figure 6:**
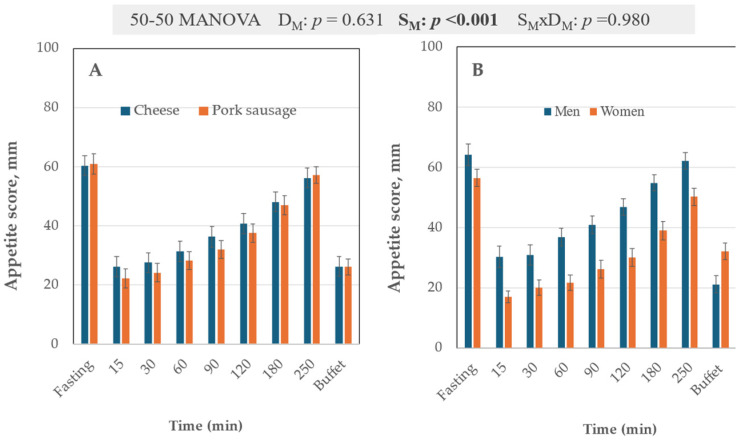
Panels (**A**,**B**) show the time profiles for the appetite scores for sex and diet. The results of the 50-50 MANOVA test for the time profile are indicated above the histograms. Error bars show mean ± s.e.m.

Appetite scores (Figure 6A,B) represent all VAS variables averaged [35]. Appetite scores (timescale data) were sex-dependent, with men expressing higher appetite scores. The appetite scores were not sensitive to diet (Figure 6A). However, the cheese diet gave numerical higher appetite scores (Figure 6A). When the variable VAS Hunger score for cheese and VAS Hunger score for pork was used, the time 90–180 min scored significantly higher (*p* < 0.026, Z sample test) for cheese, while the appetite scores did not detect any significant difference.

### 3.3. Post-Buffet VAS and Buffet Energy Intake

Post-buffet appetite scores did not clearly depend on sex (*p* = 0.083). Post-buffet VAS fullness was higher for men (79 versus 68 mm *p* = 0.008, RM-ANOVA). For the ad libitum buffet variables, sex dominated this variable, while diet had EV equal to 0.35%. Males consumed more energy than females (791 ± 64 kcal versus 344 ± 32 kcal, *p* < 0.001).

## 4. Discussion

In this study, we aimed to investigate whether two breakfast meals with different animal products (Gouda-type cheese and pork sausages) differed in their postprandial plasma appetite-related hormones, lipid responses, and subjective appetite feelings in a cohort of young individuals. Our diet discussion will be mainly focused on TG, ghrelin, GLP-1, and GIP since their diet differences were supported by both the two normalized data analyses (MN RM-ANOVA and MN 50-50 MANOVA) that identified the factors D (not for TG), D*T, and D_M_ as significant. Moreover, the cheese meal led to more sustained GLP-1 and GIP responses compared to the sausage meal. In addition, we observed that the meal effects were sometimes influenced by sex, which could suggest that these products have different effects in men and women. Thereafter, the sex variable effects will be discussed, as well as the methods used for this study.

### 4.1. Diet Effect

The overall diet factor was small. This was somewhat expected since the test products had very similar macronutrient composition, i.e., the same amount of total fat and proteins but with significant differences in fatty acid and amino acid compositions. Another important nutritional difference between meals was the variation in the content of the micronutrient calcium, which could also influence the postprandial responses. However, since we only examined two test samples, only total product differences can be quantified and used in statistics and not a specific inherent chemical component.

Our calculations could not detect average raw data differences in TG as diet-dependent. However, we observed differences in TG time curves, i.e., serum TG changes related to diet. The pork sausage meal promoted earlier serum TG reductions than the cheese diet did, thereby supporting a real D*T effect. The trend for LDL to peak at 90 min for pork sausages may reflect this earlier TG decline. Low postprandial TG responses have been found to impact cardiovascular risk, even though using post-meal TG plasma concentrations to assess the increased risk is not standardized [43]. The authors suggested that lower TG peak values and a faster return to basal values are associated with a smaller CVD risk. This is also supported by a meta-analysis investigating the effects of different fatty acids on postprandial TG levels [44]. A medium hard cheese’s TG peak may occur at 4 h or later, and TG had a shorter time above fasting level for sausages, although this could not be clearly documented from a 4 h test [45]. Finally, the use of 13 % of the dairy fat from butter may influence TG profiles.

Serum TG following a fat rich meal is determined by many factors in the digestion of the meal, such as the gastric lipases, bile acids, pH, and consistency of the foods, as well as the saturation and length of the fatty acids [45]. The higher TG peak at 3 h postprandially for the sausage test meal compared to the cheese test meal may depend on fatty acid chain length and degree of saturation. As shown by Muesing et al. [46] and Pedersen et al. [47], a tendency towards a higher degree of lipemia was observed after consumption of MUFAs compared to long-chain SFAs. This may agree with the present findings as sausages contain more MUFA compared to cheese. Tholstrup et al. [48] showed that at 4 h postprandial, plasma TG was higher after consumption of oleic acid and PUFAs than palmitic and stearic acid. Interestingly, in the present study, at 4 h, the serum TG concentration was reduced in the pork diet group compared to the cheese group, indicating that the TG clearance from the circulation may differ when consuming these two test meals. It is possible that the 4 h reduction in serum TG with the sausage test meat may be caused by higher plasma lipoprotein lipase activity 4 h after MUFA intake as reported by Tholstrup et al. [48]. Moreover, this experiment revealed substantial individual variation in lipids and leptin concentration values in the fasting stage. The small diet effect for LDL, for instance, may also reflect different basal values preventing a clear conclusion regarding the absolute values of LDL that are used clinically. 

Regarding ghrelin, the effect was that cheese induced higher remaining ghrelin (as defined by secretion minus clearance) than pork sausages, and this was also supported by VAS data (Figure 5). Theories related to the impact of essential and conditionally essential amino acids on satiety (Supplementary Materials, Table S1) support the observed changes in ghrelin [49]. Our analysis revealed that ghrelin concentrations were maintained at lower values for longer periods following the pork sausage breakfast. In support of our data, a study investigating the effects of high-fat meals on women with obesity reported a greater reduction in ghrelin following PUFA and MUFA meals compared to SFA [50]. 

Food products with modulatory effects on GLP-1 expression and secretion are of interest [51]. GLP-1 (and GIP) showed differences between the two meals which could, at least in part, be explained by amino acid composition differences and types of fatty acids. It has been described that nutrients such as calcium could also affect the secretion of these hormones [52,53]. A study conducted by Thomsen et al. [3] observed that an olive oil meal increased GLP-1 more than butter over the same time range as our study. The effects of protein and amino acids in these hormones have also been explored. A study by van der Klaauw et al. [54] showed that the influence of protein on GLP-1 release increased significantly over time, with protein dominating GLP-1 concentration values three to four hours post-meal. In addition, at 60–90 min, GLP-1 release was numerically higher for the fat meal than for the protein meal [54]. Moreover, specific amino acids such as phenylalanine, arginine, glutamine, and tryptophan have been identified as key stimulators of GLP-1 release [55]. While essential amino acids may enhance satiety, the direct relationship between specific amino acids and GLP-1 release requires further investigation. Additionally, the interaction between protein-derived amino acids and calcium may stimulate GLP-1 production, as evidenced by Mace et al. [56], who reported that increasing the calcium-to-phenylalanine molar ratio significantly enhanced GLP-1 response. Another study comparing animal products (eggs versus cottage cheese) with similar macronutrient composition detected a delay in GLP-1 secretion after the omelette meal, which agrees with our data [53]. 

Our analysis clearly linked cheese to higher active GLP-1 at 240 min. In principle, this could be possible considering the dairy amino acids available to stimulate GLP-1 secretion. The effect of calcium on GLP-1 secretion should not be ruled out either. Interestingly, in one study investigating the effects of dairy products on postprandial metabolism, all dairy except cheese increased pre-meal GLP-1 [57]. Recently, Watkins et al. [58] reported that the potential for calcium to increase GLP-1 depends on dairy protein structure/microstructure.

Conversely, the different meal effects on circulating GIP are unclear. A slower decline in plasma GIP post-peaking was, however, significant for cheese. Due to the slower decline in GIP, it was numerically higher at 240 min when the dairy product was consumed. Our MN 50:50 MANOVA data for GIP are supported by Chang et al. [59] who reported higher GIP one hour after the intake of SFA than after MUFA and PUFA meals (using milkshakes as the test products). It has been reported that the source of dietary fats affects the secretion of GIP due to different absorption in the small intestine [60]. Contrary to these findings, one study comparing the effects of various fats on GIP secretion reported lower GIP concentrations after fish oil than olive oil and corn oil, but higher GIP levels than cocoa butter [60]. Moreover, data from the Cocoheart study detected a higher GIP peak after a mix of safflower and olive oil compared to butter and coconut oil [61]. While unsaturated fats can promote a more substantial incretin response than saturated fats [60], this may also depend on the time post-test meal.

Regarding the interaction of GIP and insulin, even though higher GIP levels are shown to increase insulin secretion and promote satiety, many studies associate GIP levels with obesity. On the other hand, in people with type 2 diabetes, the incretin effect—including GIP response—is almost entirely lost [62].

Diet, VAS, and ad libitum buffet test: Our observation regarding diet and the ad libitum test was as expected and in agreement with Chang et al. [59]. The challenge with the buffet test is a presumed high clinical relevance combined with a low biological understanding of what affects it [63]. Our calculations (Supplementary Materials, Table S3) revealed that the diet effect on VAS variables was small. Flint et al. [64] suggested a criterion for significance (5 mm difference on mean VAS scores) using a paired approach and 18 subjects. In agreement with this criterion, a paired test revealed that pork sausages maintained a period of lower hunger scores and lower ghrelin concentration compared to cheese.

### 4.2. The Sex Factor

There are two types of sex effects: (i) at fasting and (ii) during the postprandial period. At fasting, leptin, HDL and glucose, with some trend for CCK and TC, appeared to be sex-dependent. Albeit the statistical methods being different, TG and GLP-1 were found to depend on sex during the postprandial period.

The TG sex difference during the postprandial phase may reflect the biological differences in lipoprotein metabolism. Notably, our findings suggest that while women showed an early peak and plateau in TG levels, men continued to exhibit elevated levels for up to 240 min. This observation aligns with the existing literature indicating that women generally have faster TG clearance rates than men [65,66]. The postprandial sex dependency for ghrelin was only supported by MN 50-50 MANOVA. However, ghrelin activation has also been reported to depend on sex [67]. Since our ghrelin differences at fasting appear smaller than those given by Leone et al. [67], their data could also be interpreted in the same way as ours, i.e., that at longer times (180–240 min), males have more ghrelin suppression. Vollmer et al. [68] reported a sex effect for GLP-1 during a postprandial study where either oral glucose or a mixed meal was ingested. 

Sex, appetite scores, clinical or energy markers, and ad libitum buffet test: Subjective appetite markers mostly reflected sex differences. Future research should consider biological sex as a critical factor in nutritional responses. Females tended to exhibit faster ghrelin suppression and lower subjective appetite scores than males. Females generally require fewer calories to reach satiety than males in agreement with our data. In addition, one study comparing the effects of different dairy products with water reported that women had lower later food intake effects compared to men [57].

Acosta et al. [63] investigated factors influencing satiation variability (meal size in ad libitum test) following a standardized breakfast given to 717 adults. The authors found sex, ethnicity, and age to explain part of the variability in satiation. Interestingly, height and fat percentage had smaller associations, and gastrointestinal hormones had a minor contribution. Their model gave a total EV of 25%, suggesting that other factors, such as genetic characteristics, may explain the larger part of satiation [63].

### 4.3. Methodology

Since the raw data acquired for blood markers was not affected by diet, an efficient normalization method was selected. Using leptin as an example, sex was a large factor at fasting and dominated as a factor during the postprandial phase. Normalization strongly reduced the magnitude of the factors S, S_M,_ and S*T, as observed for leptin, and HDL. This reduction increased the size of the diet factors for D*T and D_M_, but also for D. Scale transformation showed more clearly how the time profiles depended on design variables. However, it must be emphasized that comparisons between variables must always encompass investigating the raw data as well. 

The major disagreement between the statistical methods RM-ANOVA and 50-50 MANOVA appeared for insulin and glucose. These variables had the highest and most significant D*T, suggesting a significant and large difference between diets. This could neither be supported by 50-50 MANOVA, nor by comparing insulin/glucose with TC/HDL/LDL. The latter group of variables was of little importance for differing test diets, but more so than the insulin/glucose group. Therefore, D*T from RM-MANOVA may not alone be sufficient to identify a diet effect, especially for the insulin level that had a large change over a short time. Since the individuals’ metabolic differences in the time direction were not corrected through scale adjustment, the two statistical methods could also handle the data differently. Possibly, data like insulin, with substantial scatter and few subjects, may not be suitable for some statistical methods. In addition, the results from a few subjects, possibly also belonging to different metabolic groups, should always be taken as mostly hypothesis-generating.

MN 50-50 MANOVA rejected significance *(p* < 0.05) for smaller factors (EV% < 4) than did MN RM-ANOVA. In addition, each factor’s independent validation step in 50-50 MANOVA, i.e., through PCA scores, may identify the size and numbers of factors differently to avoid incorrect causality suggestions especially for the seven consecutive time variables. RM-MANOVA, on the other hand, is fitting data to models. Our approach, to this end, nevertheless suggested that using two different statistical approaches may be useful.

### 4.4. Limitations of the Study

The results may have been different if another age range had been analyzed.

Including PYY analysis may have provided a better satiety marker for our participants. Using time windows > 4 h could be extended to achieve more information. Taste perception data of participants may have cast more light on the outcome.

## 5. Conclusions

Our results show that two breakfast meals with distinct animal products elicited postprandial differences in appetite-related hormones, lipids, and subjective appetite feelings over a period of 4 h. A slower triglyceride clearance following cheese consumption was observed. This aligns with the existing literature, suggesting that the composition of fatty acids plays a significant role in regulating postprandial lipid metabolism. Furthermore, ghrelin suppression was prolonged after the pork meal, indicating that appetite control may be impacted differently by variations in fatty acids and amino acids. In contrast, the cheese meal, characterized by a higher saturated fat content and calcium, resulted in more sustained responses of GLP-1 and GIP compared to the sausage meal. The contrasting results for ghrelin and GIP/GLP-1 (favouring the diets differently) could not be linked to differences in promoting satiety at 4 h.

## Figures and Tables

**Table 1:** Content and macronutrient distribution of the breakfast meals.

Test Meal	Kcal *	Orange Juice (g)	Bread (g)	Tomatoes (g)	Butter (g)	Test Product (g)	Macronutrients (E%) **		
							Fat	Carb.	Protein
Cheese	598	184	69	23	4.8 ***	104 (27% fat)	46.5	32.0	21.5
Pork sausage	596	184	69	23	-	152 (21% fat)			

* Example is based on 2000 kcal/day diet; ** E%: energy percentage; *** 17% water.

**Table 2:** Macronutrient distribution and energy content of the cheese and sausage breakfasts per 100 g.

	Carbohydrates (g)	Protein (g) ^#^	Fat (g) ^#^	Energy (kJ/kcal) ^#^
Cheese	0	27	27	1458/351
Sausage	0	20	21	1127/269

^#^ *p* < 0.05—Significant differences between breakfast meals.

**Table 3:** Fatty acid composition in the test products (g fatty acid in total test product); see weights in Table 1). The total fat of the test product was 32 g. Daily intake of 2000 kcal is used in this example.

Fat Source	C4:0 (g)	C6:0–C12:0 (g)	>C12:0 (g)	Sum SFA (g)	Sum * MUFA (g)	Sum C18:1tr (g)	Sum CLA (g)	Sum PUFA (g)	Unidentified (g)	n6:n3 Ratio
Pork fat	<LOQ **	0.03 ± 0.02	10.31 ± 0.06	10.34 ± 0.06	14.22 ± 0.27	0.07 ± 0.009	<LOQ	5.17 ± 0.23	0.88 ± 0.10	7.9 ± 0.3
Dairy fat ***	1.15 ± 0.02	3.13 ± 0.003	16.73 ± 0.003	21.02 ± 0.033	6.54 ± 0.006	0.38 ± 0.003	0.10 ± 0.003	0.69 ± 0.009	1.78 ± 0.009	3.05 ± 0.02

* MUFA is calculated without trans; ** LOQ = level of quantification; All fatty acid groups were significantly different (*p* < 0.05, *t*-test, double-sided) between pork and dairy fat. *** includes butter fat.

**Table 4:** Selected amino acid (AA) (mean ± s.e.m.) * present in test products and bread **. Other amino acids and details [31,32] can be found in Supplementary Materials (Table S1). Daily intake of 2000 kcal is used in this example.

Amino Acid	Wheat and Rye Bread (g/Test Diet)	Pork Sausage (g/Test Diet)	Gouda Cheese (g/Test Diet)	p ***
Tryptophan	0.085 ± 0.003	0.334 ± 0.007	0.289 ± 0.008	0.053
Phenylalanine	0.401 ± 0.006	0.985 ± 0.002	1.327 ± 0.056	0.026
Arginine	0.334 ± 0.001	1.832 ± 0.028	1.750 ± 0.004	<0.001
Glutamate and Glutamine	3.252 ± 0.036	4.553 ± 0.067	6.389 ± 0.015	<0.001 ****

* Mean ± s.e.m, (standard error of the mean); ** Tomato protein was ~1.3% of total protein in test diet but is not included in the calculations; *** pork sausage versus cheese tested at the same total AA, *t*-test, double-sided; **** Co-determined due to acid hydrolysis.

**Table 5:** Baseline characteristics of the participants (n = 24).

Anthropometric Variables	Values
Sex (number of men/women)	13/11
BMI (kg/m^2^)	24.5 ± 2.6
WC (cm)	82.4 ± 8.8
SBP (mmHg)	125.5 ± 11.8
DBP (mmHg)	79.0 ± 6.7
**Appetite markers**	**Values**
Ghrelin (pmol/L)	44.2 ± 21.2
GIP (pmol/L)	8.3 ± 6.7
GLP-1 (pmol/L)	30.0 ± 10.2
CCK (pmol/L)	0.89 ±0.67
Leptin (pmol/L)	2354 ± 2159
**Clinical markers**	**Values**
Insulin (pmol/L)	46.4 ± 17.7
LDL (mmol/L)	2.62 ± 0.63
HDL (mmol/L)	1.48 ± 0.35
Total cholesterol (mmol/L)	4.35 ± 0.72
Triglycerides (mmol/L)	0.96 ± 0.32

Data presented as mean ± s.d.; WC = Waist circumference, SBP = Systolic blood pressure; DBP = Diastolic blood pressure.

## Data Availability

Fully anonymized data can be made available through the corresponding author from 1 July 2025.

## Supplementary Materials

The following supporting information can be downloaded at: https://www.mdpi.com/article/10.3390/nu17091581/s1, Text S1: Sausage production; Figure S1: Figures of time profiles; Table S1: Amino acid composition; Table S2: RM-ANOVA statistics; Table S3: 50-50 MANOV statistics; Table S4: Raw data measured at different times.

## Abbreviations

The following abbreviations are used in this manuscript:

ANOVA	Analysis of variance
CCK	Cholecystokinin
GLP-1	Glucagon-like peptide-1
GLM	Generalized linear model
GIP	Glucose-dependent insulinotropic polypeptide
HDL	High-density lipoprotein
LDL	Low-density lipoprotein
MANOVA	Multivariate analysis of variance
MN	Max normalized
MUFA	Monounsaturated fatty acid
PCA	Principal component analysis
PLSR	Partial least squares regression
PUFA	Polyunsaturated fatty acid
PYY	Peptide YY
RM-ANOVA	Repeated measure analysis of variance
SFA	Saturated fatty acids
VAS	Visual analogue scale
TC	Total cholesterol
TG	Triglycerides
