# Supplementary material for: Postprandial Responses to Animal Products with Distinct Fatty Acid and Amino Acid Composition Are Diet-Dependent
Source: Nutrients. 2025 May 4;17(9):1581. doi: 10.3390/nu17091581 (PMC12073875; doi:10.3390/nu17091581)
Supplement: Supplementary file 1 [file nutrients-17-01581-s001.zip › R1 Supplementary 28 04 2024.pdf]

SUPPLEMENTARY

Text S1 Sausage production

The following recipe was used: 100 g sausage mince, 1 g NaCl, 0.8 g black pepper, 0.5 g salvia, 0.5 g coriander and 0.8 g nutmeg. The blending of ingredients (1.4 min) was first performed and then chopped for 5 min with the temperature kept below 10°C (Chopper KU 65AC, LASKA Makartstraße 60A-4050 Traun/Austria). The spiced sausage batter was put in the natural casing (pork) and heated in a Doleschal heat cabinet SC 2000 (Program: 10 min up, 25 min to 78 °C, 97% humidity used, air venting for 5 min, 15 min for chilling, then showering, total time used was 60 min). The weight loss during processing was 13%. The breakfast sausage was produced in a pilot plant authorized for human food production (Nofima, Ås, Norway). The heat-treated sausages were frozen at -20°C. Then, they were thawed and microwaved on the serving plate to an internal sausage temperature of (60°C).

Table S1 Amino acid composition

Table S1. Amino acid (AA) composition; g amino acid (mean ± s.e.m.)*; provided from each test product and bread** Daily intake of 2000 kcal is used in this example.

Amino acid [A]	From wheat & rye bread (g/test diet)	From pork sausage (g/ test diet)	From Gouda cheese (g/ test diet)	*p-value* (pork versus cheese diet at the same total AA)	Adult DRI (mg/ day* 70 kg) (g/ day)[B]	"Bliss" point, g /day[C]
Tryptophan, Trp, W	0.085±0.003	0.334±0.007	0.289±0.008	0.053	0.35	0.75
Cysteine, Cys, C; Cond ess.	0.149±0.001	0.294±0.006	0.109±0.005	0.002		0.9
Methionine, Met, M	0.115±0.002	0.762±0.009	0.762±0.021	0.999	1.33	1.4
Histidine, His, H	0.172±0.001	1.153±0.017	0.909±0.015	0.009	0.98	1.9
Tyrosine, Tyr, Y; Cond.ess.	0.075±0.024	0.649±0.003	0.965±0.065	0.049		2.1
Phenylalanine, Phe, F	0.401±0.006	0.985±0.002	1.327±0.056	0.026	2.31	2.6
Threonine, Thr, T	0.235±0.004	1.369±0.001	1.082±0.013	0.019	1.40	2.6
Isoleucine, Leu, L, BCAA	0.299±0.001	1.396±0.003	1.425±0.019	0.256	1.33	3.1
Valine, Val, V, BCAA	0.336±0.001	1.313±0.019	1.523±0.034	0.011	1.68	3.2
Lysine, Lys, K	0.172±0.004	2.727±0.001	2.422±0,019	0.004	2.66	4.6
Leucine, Leu, L, BCAA	0.551±0.001	2.340±0.004	2.726±0.029	0.008	2.94	4.7
Arginine, Arg, R	0.334±0.001	1.832±0.028	1.750±0.004	<0.001		
Glycine, Gly, G	0.258±0.003	1.603±0.051	0.435±0.008	0.002		
Proline, Pro, P	0.884±0.017	1.611±0.034	2.957±0.015	0.001		
Serine, Ser, S	0.378±0.001	1.183±0.001	1.392±0.019	0.004		
Alanine, Ala, A	0.235±0.003	1.674±0.007	0.740±0.003	<0.001		
Aspartate, Asp, D	0.348±0.004	2.392±0.040	1.763±0.006	0.004		
Asparagine, Asn, N				Codetermined due to acid hydrolysis		
Glutamate, Glu, E and	3.252±0.036	4.553±0.067	6.389±0.015	<0.001		
Glutamine, Gln, Q				Codetermined due to acid hydrolysis		
Asparagine, Asn, N	-			Not determined		
Glutamine, Gln, Q				Not determined		

*Mean ±s.e.m, (standard error of the mean); **Tomato protein was ˜1.3% of total protein in test diet but is not included in the calculations. [A]) Red = essential and yellow = conditionally essential (FAO/WHO/UNO, 2002). [B]) Hou and Wu, 2018, Kendall, 2024). Intake < bliss increases craving.

Table S2 RM-ANOVA statistics

Table S2. Repeated measure ANOVA with, within and between, subject effects.

	Within subjects										Between subjects	
Variables	Diet		Time		Diet*Time		Diet*Sex		Sex*Time		Sex	
	**ETA**	***p***	**ETA**	***p***	**ETA**	***p***	**ETA**	***p***	**ETA**	***p***	**ETA**	***p***
TG-22**	0.023	0.478	0.444	**<0.001**	0.089	0.111	0.00	0.948	0.156	**0.029**	0.014	0.910
TG-MN-AN	0.058	0.258	0.275	**<0.001**	0.482	**<0.001**	0.005	0.740	0.077	0.137	0.076	0.192
TC-22	0.035	0.378	0.108	**0.049**	0.029	0.584	0.007	0.702	0.039	0.441	0.082	0.175
TC-MN-AN	0.188	**0.034**	0.184	**<0.001**	0.336	**<0.001**	0.004	0.773	0.111	**0.046**	0.020	0.847
HDL-22	0.009	0.653	0.145	**0.024**	0.036	0.481	0.023	0.482	0.079	0.14	0.153	0.059
HDL-MN-AN	0.00	0.978	0.053	0.291	0.136	0.614	0.001	0.887	0.041	0.436	0.020	0.763
LDL-22	0.015	0.568	0.134	**0.028**	0.042	0.410	0.080	0.180	0.021	0.703	0.029	0.430
LDL-MN-AN	0.001	0.901	0.065	0.198	0.131	**0.005**	0.010	0.642	0.009	0.976	0.015	0.563
Leptin-22	0.063	0.235	0.368	**<0.001**	0.045	0.369	0.194	**0.031**	0.135	**0.039**	0.500	**<0.001**
Leptin-MN-AN	0.066	0.224	0.437	**<0.001**	0.512	**<0.001**	0.019	0.517	0.008	0.98	0.015	**0.189**
Ghrelin-22	0.012	0.613	0.582	**<0.001**	0.024	0.672	0.008	0.686	0.069	0.171	0.008	**<0.001**
Ghrelin-MN-AN	76.1	**<0.001**	0.762	**<0.001**	0.761	**<0.001**	0.028	0.431	0.029	0.692	0.029	0.430
GLP-1-22	0.009	0.653	0.726	**<0.001**	0.027	0.642	0.012	0.610	0.112	**0.041**	0.079	0.185
GLP-1-MN-AN	0.759	**<0.001**	0.599	**<0.001**	0.525	**<0.001**	0.00	0.925	0.077	0.128	0.003	0.815
GIP -22	0.010	0.646	0.627	**<0.001**	0.013	0.840	0.002	0.826	0.024	0.688	0.008	0.677
GIP-MN-AN	0.35	**0.002**	0.541	**<0.001**	0.682	**<0.001**	0.061	0.244	0.059	0.230	0.003	0.474
CKK-22	0.007	0.706	0.634	**<0.001**	0.060	0.233	0.066	0.226	0.072	0.162	0.018	0.53
CKK-MN-AN	0.109	0.115	0.269	**<0.001**	0.505	**<0.001**	0.182	**0.037**	0.081	0.08	<0.01	0.952
Glucose-22	0.004	0.781	0.761	**<0.001**	0.029	0.579	0.012	0.617	0.035	0.480	0.060	0.248
Glucose-MN-AN	0.796	**<0.001**	0.656	**<0.001**	0.636	**<0.001**	0.010	0.645	0.036	0.556	0.014	0.577
Insulin-22	0.062	0.239	0.774	**<0.001**	0.047	0.353	0.008	0.686	0.055	0.279	<001	0.922
Insulin-MN-AN	0.895	**<0.001**	0.774	**<0.001**	0.727	**<0.001**	0.279	0.06	0.063	0.190	0.031	0.408

*Model used for within Subjects Design: timepoints + diet + timepoints * diet + timepoint *sex+ timepoint*diet*sex. Higher order result was not included; it was only significant for TC (P = 0.023) and insulin (MN version, P=0.046). Bold letters mean P< 0.005.

** Absolute (raw)values: *** Input data was MN. # ETA (Partial ETA squared) means the proportion of variance explained by a variable in an ANOVA model after accounting for other variables.

Figure S1- Figures of time profiles

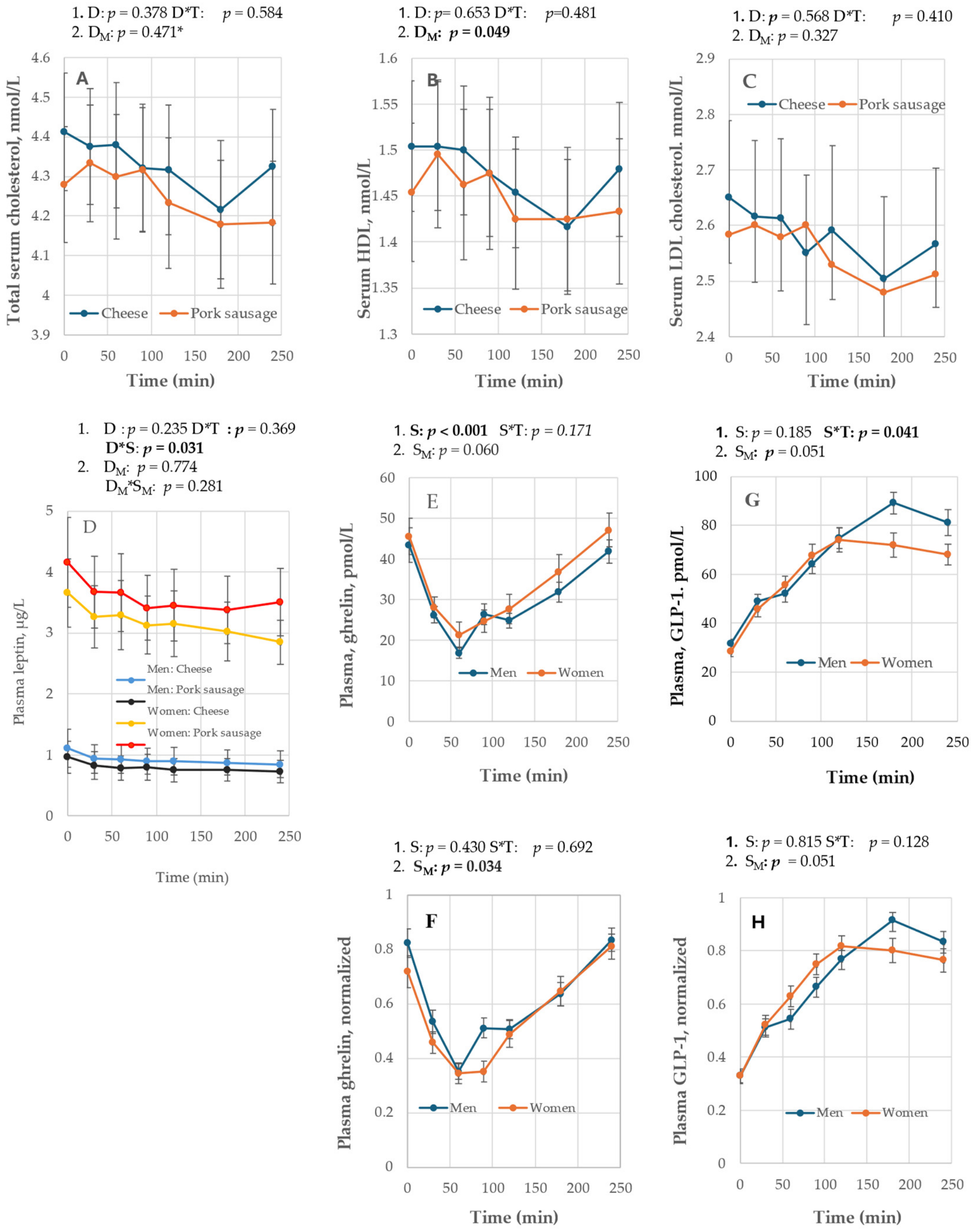

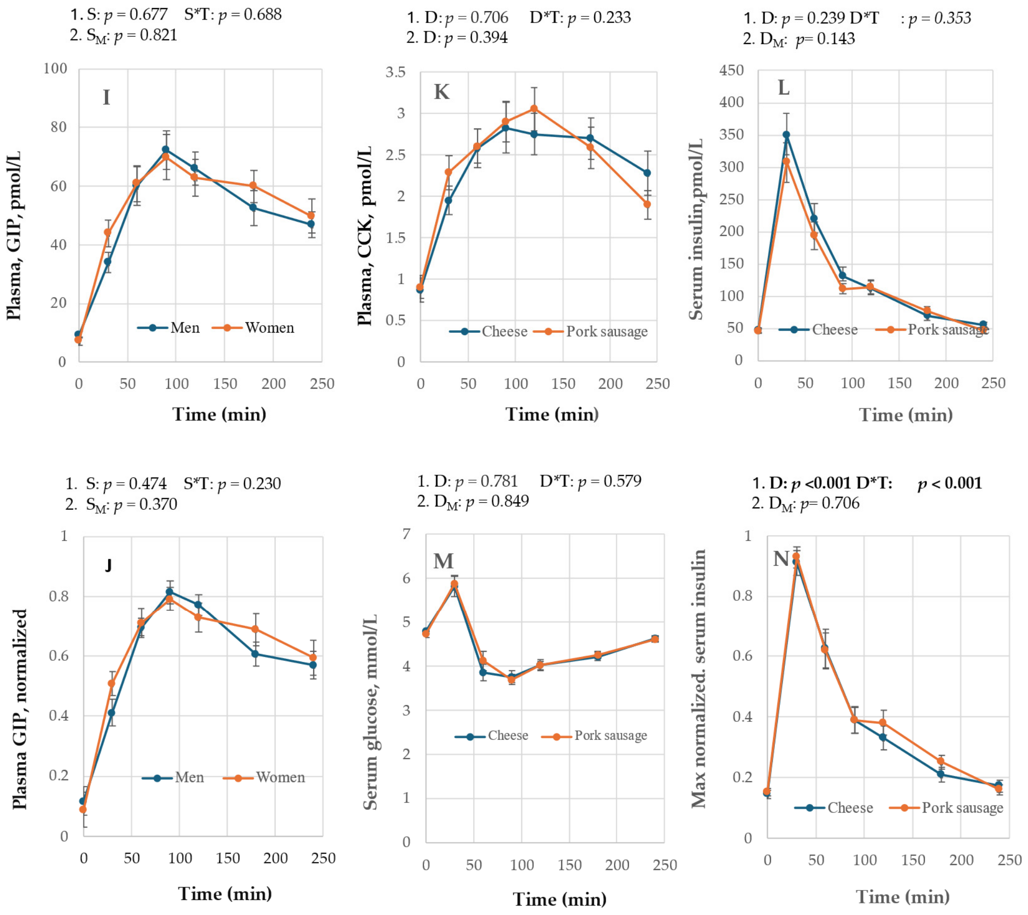

Figure S1. Measured data split on the two diets (panel **A-C, K**). Panel **D** is split on both diet and sex. Panels **E- J** show normalized data for ghrelin, GLP-1 and GIP split on 2 separate panels regarding diet and sex. Panels **L** and **N** provide both absolute and normalized panels of insulin, respectively. Above panels, the *P*-values of key factors being outputs from the statistical models (MN) RM ANOVA (1) and (MN) 50-50 MANOVA (2), are listed. Abbreviations: D, D_M, S and S_M are diet and sex factors used in RM-ANOVA and 50-50 MANOVA, respectively. T indicates the time factor. Mean and s.e.m. were used for data in figures. Bold letters mean $p < 0.05$.

Table S3 50-50 MANOVA statistics

Table S3. Explained variance (EV) and *p* value from 50-50 MANOVA with or without normalized (MN) time profiles using absolute (raw) and normalized time profiles

Variables		% Explained variance *(EV) and *P*			
		**Sex**	**Diet**	**Sex* Diet**	**Model error**
TG	EV%	4.8 *(7.6)* **	1.1 *(7.5)*	0.3 *(1.4)*	91.8 *(72.5)*
	p	**0.002 *(<0.001)***	0.123 ***(0.004)***	0.398 *(0.496)*	
TC	EV%	5.6 *(0.9)*	0.37 *(0.9)*	0.09 *(0.5)*	89.6 *(94.9)*
	p	0.471 *(0.651)*	0.897 *(0.758)*	0.966 *(0.896)*	
HDL	EV%	10.5 *(0.8)*	0.20 *(0.9)*	0.29 *(0.5)*	83.2 *(94.9)*
	p	**0.049** *(0.414)*	0.968 *(0.941)*	0.657 *(0.771)*	
LDL	EV%	2.4 *(0.5)*	0.14 *(1.60)*	0.52 *(0.9)*	96.5 *(93.9)*
	p	0.327 *(0.920)*	0.855 *(0.739)*	0.645 *(0.869)*	
Leptin	EV%	48.4 *(5.1)*	1.4 *(2.6)*	1.3 *(0.3)*	48.9 *(80.7)*
	P	**<0.001** *(0.121)*	0.774 *(0.310)*	0.281 *(0.961)*	
Ghrelin	EV%	4.9 *(3.9)*	1.0 *(4.0)*	1.3 *(2.4)*	88.3 *(89.2)*
	P	0.060 ***(0.034)***	0.780 ***(0.040)***	*0.767 (0.167)*	
GLP-1	EV%	3.7 *(3.6)*	*1.2 (12.1)*	1.4 *(1.1)*	88.7 *(81.9)*
	P	0.051 ***(0.022)***	*0.629* ***(<0.001)***	*0.383 (0.815)*	
GIP	EV%	1.0 *(2.2)*	0.49 *(8.3)*	1.0 *(3.6)*	92.2 *(83.0)*
	P	0.821 *(0.370)*	0.963 ***(0.001)***	0.584 *(0.127)*	
CCK	EV%	3.3 *(2.3)*	*1.2 (2.5)*	0.8 *(1.3)*	88.6 *(84.9)*
	p	**0.048** *(0.269)*	0.394 *(0.372)*	0.775 *(0.784)*	
Glucose	% EV	1.8 *(1.2)*	0.7 *(1.1)*	1.1 *(2.7)*	87.9 *(91.5)*
	P	0.376 *(0.819)*	0.849 *(0.513)*	0.396 *(0.437)*	
Insulin	EV%	10.5 *(1.3)*	2.2 *(1.3)*	2.4 *(1.9)*	84.1 *(89.6)*
	P	**0.028** *(0.623)*	0.143 *(0.706)*	0.117 *(0.896)*	
VAS Hunger, (7 time points)	EV%	13.4 *(9.1)* **	2.0 *(1.8)*	0.9 *(2.8)*	83.7 *(86.2)*
	p	**0.013 (0.037)**	0.545 *(0.425)*	0.430 (0.180)	
VAS Fullness, (7 time points)	EV%	9.0 *(9.1)*	0.2 *(0.3)*	1.0 *(1.2)*	89.8 *(88.2)*
	p	**0.023 (0.021)**	0.676 *(0.963)*	*0.579 (0.560)*	
3 hunger, (3 VAS averaged-fullness), (7 time points)	EV%	20.2 *(7.8)*	1.3 *(1.8)*	0.6 *(1.6)*	77.9 *(88.8)*
	p	**0.001 (*0.052*)**	0.592 *(0.408)*	0.942 (0.373)	
Appetite score, (4 VAS averaged), (7 time)	EV%	20.4 *(7.8)*	1.0 *(1.0)*	0.6 *(1.0)*	78.0 *(88.8)*
	P	**<0.001** *(0.066)*	0.619 *(0.703)*	0.881 *(0.411)*	
VAS Fullness post buffet***	EV% (*p*)	**13.5 (0.008)**	n.a	n.a	85.6
Buffet intake (g)***	EV% (*p*)	**43.2 (0.001)**	<0.1 (0.901)	0.39 (0.695)	56.4
Buffet intake (kcal)***	EV% (*p*)	**42.8 (< 0.001)**	<0.1 (0.980)	0.35 (0.586)	57.0

*Equals Adjusted Sum of square, R^2 **Italic numbers in parentheses are calculated using normalized data; ***Normalization is not relevant. Bold numbers are significant (p > 0.05)

Table S4 Raw data measured at different times

Table S4. Raw (absolute values) changes in selected blood variables with time (mean and s.e.m.)

	Basal	Time after test meal (min)					
Variable	**0**	**30**	**60**	**90**	**120**	**180**	**240**
TG	0.96	0.98	1.10	1.15	1.27	1.40	1.39
(mmol/L	±0.04	±0.04	±0.05	±0.05	±0.07	±0.09	±0.10
TC	4.34	4.35	4.34	4.32	4.28	4.20	4.25
(mmol/L	±0.10	±0.10	±0.11	±0.11	±0.11	±0.12	±0.10
HDL	1.48	1.50	1.48	1.48	1.44	1.42	1.46
(mmol/L	±0.07	±0.07	±0.08	±0.08	±0.07	±0.07	±0.08
LDL	2.63	2.63	2.61	2.59	2.57	2.51	2.55
(nmol/L	±0.09	±0.09	±0.10	±0.09	±0.10	±0.09	±0.09
Leptin	2353	2066	2058	1956	1957	1906	1880
(pmol/L)	±308	±265	±276	± 247	± 260	±245	±238
Ghrelin	44.0	27.2	19.7	24.5	27.8	36.1	45.2
(pmol/L)	±3.0	±1.6	±1.6	±1.9	±2.0	±2.4	±2.7
GLP-1	30.0	47.3	53.6	65.6	74.1	80.9	74.8
(pmol/L)	±1.4	±2.1	±2.5	±3.0	±3.2	±3.4	±3.6
GIP	8.4	38.6	60.4	71.2	64.5	55.9	48.2
(pmol/L)	±0.8	±2.8	±4.5	±4.9	±4.1	±4.1	±3.5
CCK	0.89	2.12	2.59	2.86	2.90	2.64	2.09
(pmol/L	±0.10	±0.13	±0.15	±0.19	±0.18	±0.17	±0.16
Insulin	46.6	328.6	206.7	121.4	113.2	72.8	50.8
(mol/L)	±2.6	±23.1	±16.3	±7.6	±8.1	±4.4	±2.7
Glucose	4.76	5.86	3.99	3.72	4.03	4.24	4.62
(mmol/L)	±0.32	±0.98	±1.02	±0.62	±0.56	±0.44	±0.31
